# Intestinal Development Patterns and Gut Microbiota Colonization Dynamics in Sichuan Bream (*Sinibrama taeniatus*)

**DOI:** 10.3390/ani15233431

**Published:** 2025-11-28

**Authors:** Shixia Huang, Qiang Zhao, Chao Deng, Yuexin Sun, Xiao Yang, Shilin Li, Tianzhi Jin, Zhe Zhao, Kaixuan Liu, Qilin Feng, Hailong Ge, Zhijian Wang, Fang Li

**Affiliations:** 1 Integrative Science Center of Germplasm Creation in Western China (Chongqing) Science City, Southwest University, Chongqing 401329, China; sesia1998@outlook.com (S.H.); luckzhao0823@163.com (Q.Z.); 19556272290@163.com (Y.S.); yangxiaosci@outlook.com (X.Y.); lslpgc@163.com (S.L.); jtzswu@foxmail.com (T.J.); 18883779783@163.com (Z.Z.); 18359461500@163.com (K.L.); 13216335633@163.com (Q.F.); gehailong1992@126.com (H.G.); 2 Key Laboratory of Freshwater Fish Reproduction and Development (Ministry of Education), School of Life Sciences, Southwest University, Chongqing 400715, China; dc17749941501@163.com; 3 College of Fisheries, Southwest University, Chongqing 401329, China

**Keywords:** *Sinibrama taeniatus*, gut microbiota, histology, enzymology, aquatic animals

## Abstract

Sichuan bream (*Sinibrama taeniatu*), an endemic small economic fish in the upper Yangtze River, has achieved captive breeding breakthroughs. To advance standardized and large-scale aquaculture, this study investigated intestinal development, enzyme activities, gut microbiota, and its interactions with rearing water microbiota across six developmental stages (G1–G6) from hatching to sexual maturity of Sichuan bream. Results showed its progressive refinement of external morphology and intestinal developing into multiple flexures. Histology examination revealed four foregut layers with continuously increasing villus height. Digestive enzyme analysis revealed significantly higher activity of trypsin compared to that of amylase and lipase (*p* < 0.05). 16S rRNA sequencing identified Proteobacteria, Firmicutes, Bacteroidota, Actinobacteriota, and Fusobacteria as the dominant phyla in both gut and rearing water microbiota. Gut communities shifted from Proteobacteria-dominance (C_1_–C_5_) to Firmicutes-dominance (C6). The microbial source tracking analysis indicated water contributed to gut microbiota of Sichuan bream, particularly during G3. This study clarified the intestinal development patterns and gut microbiota colonization dynamics of Sichuan bream. The findings provide reference materials for the research on the growth patterns and host–microbe interaction of Sichuan bream. They lay a theoretical foundation for the protection and utilization of Sichuan bream resources, ultimately aiding in their proliferation and release.

## Simple Summary

Sichuan bream (*Sinibrama taeniatu*) is an endemic economic fish in the upper Yangtze River. To advance its standardized and large-scale aquaculture, this study investigated its intestinal development patterns and gut microbiota colonization dynamics from hatching to sexual maturity. Results showed that the intestine developed multiple flexures. Digestive enzyme analysis revealed significantly higher trypsin activity than that of amylase and lipase (*p* < 0.05). The gut microbiota shifted from Proteobacteria-dominance to Firmicutes-dominance during development, and the microbial source tracking analysis showed rearing water contributed to gut microbiota. This study clarifies the intestinal development patterns and microbiota colonization of Sichuan bream, providing a theoretical foundation for its resource protection and development utilization.

## 1. Introduction

In recent years, the gut microbiota of vertebrates has become a pivotal research focus in life sciences. Functioning as a unique ecosystem that interacts bidirectionally with its host, the gut microbiota is often termed the host’s “externalized organ”. As ancient vertebrates, fish and their gut microbiota have garnered significant attention. Fish establish their gut microbiota after hatching. During embryonic stages, microbial colonization occurs on the fertilized egg surface [1]. At this stage, the fish digestive system is not fully developed, with gut colonization commencing only after hatching [2,3]. Throughout fish development, the gut microbiota performs multifaceted functions, including nutrient absorption [4] and immune modulation [5]. Gut microbiota can participate in the digestion process of host nutrients by producing exogenous digestive enzymes, thereby promoting the growth of fish [6]. As a primary immunological barrier [7], gut microbiota combats pathogen invasion [8].

The structure of the gut microbiota shifts during fish development [9], and is influenced by multiple factors. As the fish’s survival environment, the rearing water may provide bacterial resources and influence the gut microbiota [10,11]. Additionally, studies have shown that the host’s developmental stage is an important factor influencing the gut microbiota [3,12,13]. Xiao et al. [14] discovered that genetic-mediated host selection might be an important process for the colonization of the core gut microbiota in zebrafish from larvae to adult, and the developmental stage of the fish largely influenced the succession of the gut microbial community.

Sichuan bream (*Sinibrama taeniatu*), belonging to the order Cypriniformes, family Cyprinidae, and subfamily Cultrinae, is a small economic fish endemic to the upper Yangtze River. It exhibits rapid growth, superior flesh quality, and high nutritional value, positioned as a species with significant aquaculture potential [15]. However, in recent years, due to the destruction of their habitats, the population of the Sichuan bream has sharply declined. In 2014, it was listed as a key species for artificial stocking and release by Sichuan Province. To better conserve Sichuan bream germplasm resources and enable rational exploitation, our laboratory has initiated domestication and artificial propagation of Sichuan bream in 2016. To date, breakthroughs have been made in the indoor breeding techniques for Sichuan bream. To advance standardized and large-scale aquaculture, elucidating growth patterns of Sichuan bream is essential. Previous research on Sichuan bream has focused on taxonomy and phylogeny [16], environmental adaptation [17], and reproductive regulation [18]. Furthermore, our laboratory has previously characterized the early development patterns of Sichuan bream under controlled culture conditions and established a corresponding staging system [19]. However, the patterns of intestinal development and the dynamics of microbial colonization in the gut of Sichuan bream remain unclear.

Therefore, to clarify the intestinal development pattern, the gut microbiota colonization dynamic of Sichuan bream and its relationship with rearing water, we conducted a comprehensive analysis. This analysis included data on morphology, histology, digestive/immune enzyme activity, gut microbiota, and rearing water microbiota across six developmental stages, from hatching to sexual maturity. The results will help elucidate the features of intestinal development, variations in digestive/immune enzyme activities, the dynamics of gut microbial colonization, and the contribution of rearing water to the gut microbiota of Sichuan Bream. The findings will provide reference materials for the research on the growth patterns and host–microbe interaction of Sichuan bream. They will also lay a theoretical foundation for the protection and utilization of Sichuan bream resources, ultimately aiding in their proliferation and release of Sichuan bream.

## 2. Materials and Methods

### 2.1. Hatching and Cultivating of Sichuan Bream

Broodstock of Sichuan bream, long-term domesticated for year-round reproduction ability in our laboratory, were selected for induced spawning. To ensure the quality of the data, fertilized eggs from the same batch were distributed into three aquaria as parallel groups for hatching and rearing. During this period, water temperature was maintained at 25.0 ± 0.5 °C with dissolved oxygen maintained >7.0 mg/L under a 14L:10D photoperiod. The stocking density was adjusted and gradually reduced according to the growth of the fish. As the fish grew, sequential sampling was conducted at different developmental stages. Larvae initiated first feeding approximately 2 days post-hatching. They were fed freshly hatched *Artemia* nauplii ad libitum 2–3 times daily. After 15 days of *Artemia* feeding, diet transition commenced using a specialized micro-particle formulated feed (Shandong ShengSuo Co., Ltd., Yantai, China; feed composition: imported special fish meal, Antarctic krill meal, refined fish oil, lecithin, taurine, choline chloride, vitamins and vitamin-like substances, and complex mineral elements; proximate analysis (%): crude protein ≥ 52, crude fat ≥ 8, crude ash ≤ 16.5, crude fiber ≤ 3, calcium ≤ 5, total phosphorus ≥ 1.0, and lysine ≥ 2.5) to gradually replace *Artemia*. After feeding for 30 min, feces and leftover food were removed, and one-third of the water in the aquaria was replaced. The aquaria were equipped with a recirculating water system.

### 2.2. Sample Collection

**Figure 1:**
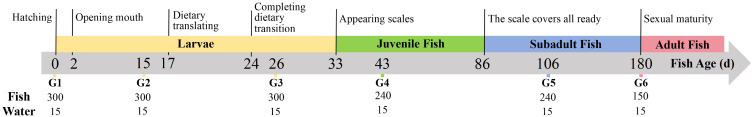
Schematic diagram of developmental period of Sichuan bream. The important host development events and husbandry events are displayed at the top. The days after hatching appear on the gray bar. The “Fish” row shows the number of fish sampled at each time point. The “Water” row shows the number of water samples (filtered by membranes) sampled at each time point.

Based on the developmental staging of Sichuan bream established by our research group [20], sampling was conducted at six critical developmental timepoints: G1 (0 days post-hatching, newly hatched larvae that have not yet opened their mouths), G2 (15 days, larvae feeding on *Artemia* nauplii), G3 (26 days, larvae completing diet transition to formulated feed), G4 (43 days, juvenile fish), G5 (106 days, subadults), and G6 (180 days, adults at first sexual maturity) (Figure 1). All experimental fish underwent 24 h fasting before sampling.

After anesthesia, external morphology and intestinal structures of the experimental fish were documented using a Canon PowerShot SH60 HS camera (Canon, Tokyo, Japan) or Nikon SMZ25 stereomicroscope (Nikon, Tokyo, Japan). Whole fish (G_1_–G_3_) or the middle section of the foregut (G_4_–G_6_) were collected for histological examination. 20 samples per stage from each group were fixed in 4% paraformaldehyde to ensure sufficient samples for observation and statistics, accounting for potential sample loss. Gut samples for enzymatic assays and microbiota sequencing (whole fish for G1) were concurrently processed. Prior to sampling, fish were rinsed with sterile water, followed by surface disinfection with 75% ethanol to obtain whole-fish samples. Subsequently, the fish were dissected using a sterile scalpel to collect intestinal samples. To meet the requirements for subsequent assays, achieving a sufficient sample size, at each time point, 40 samples (G1, G2 and G3), 30 samples (G4 and G5) and 15 samples (G6) were collected and pooled, respectively, from each replicate aquaria for enzymatic activity assays. Simultaneously, samples for microbiota sequencing were collected and pooled using the same protocol. On the same day, samples of the rearing water for microbiota analysis were collected. Water samples were collected in sterile bottles (five 1 L samples from each of the three aquaria per timepoint), and vacuum-filtered through 0.45-μm membranes. All samples for enzymatic activity, gut microbiota (including the whole-fish microbiota from G1 period), and water microbiota analyses were collected flash-frozen in liquid nitrogen and stored at −80 °C. Gut and water microbiota groups were designated C1–C6 and W1–W6, respectively, corresponding to developmental stages G1–G6.

### 2.3. Histological Examination of Intestinal Tissue

Samples fixed in 4% paraformaldehyde were dehydrated through an ethanol gradient, cleared in xylene, embedded in paraffin, and sectioned transversely at 6 μm thickness (whole-body cross-sections prepared for G1–G3 stages; intestinal cross-sections for G4–G6 stages). After hematoxylin-eosin (HE) staining, sections were mounted with neutral balsam, then examined and photographed under a Nikon upright microscope (ECLIPSE Ni-L). Morphometric analysis included villus height and width measurements, with five intact villi per section quantified. Villus height was measured vertically from the apical tip to the basal crypt, while width represented the mean of the widest and narrowest regions of individual villi [20].

### 2.4. Assays of Digestive and Immune Enzyme Activities

All enzymatic activities were determined using commercial kits. Amylase (AMS), lipase (LPS), alkaline phosphatase (AKP), acid phosphatase (ACP), and superoxide dismutase (SOD) activities were measured with kits from Nanjing Jiancheng Bioengineering Institute, Nanjing, China. Trypsin activity was assessed using kits from Suzhou Keming Biotechnology Co., Ltd., Suzhou, China. Procedures strictly followed the reagent kit manual.

### 2.5. 16S rRNA High-Throughput Sequencing

Gut samples were subjected to DNA extraction using the TIANamp Stool DNA Kit (TIANGEN BIOTECH, Beijing, China), while DNA from the filter membranes of water samples was extracted using the CTAB method. Qualified samples were subjected to PCR amplification, targeting the V3–V4 hypervariable regions with primers 515F and 806R. All PCR reactions were carried out with 15 µL of Phusion^®^ High-Fidelity PCR Master Mix (New England Biolabs, Ipswich, MA, USA); 0.2 µM of forward and reverse primers, and about 10 ng template DNA. Thermal cycling consisted of initial denaturation at 98 °C for 1 min, followed by 30 cycles of denaturation at 98 °C for 10 s, annealing at 50 °C for 30 s, and elongation at 72 °C for 30 s and 72 °C for 5 min. Mix same volume of 1× loading buffer (contained SYB green) with PCR products and operate electrophoresis on 2% agarose gel for detection. The PCR products were mixed in equimolar ratios and then purified. Sequencing libraries were generated and indexes were added. The library was checked with Qubit and real-time PCR for quantification and bioanalyzer for size distribution detection. Quantified libraries were pooled and sequenced on Illumina platforms, according to effective library concentration and data amount required.

### 2.6. Data Processing and Statistical Analysis

Raw sequences from high throughput sequencing were demultiplexed, trimmed of barcodes/primers, and merged using FLASH (Version 1.2. 11). Quality-controlled Clean Tags were chimera-filtered to obtain effective sequences. Denoising was performed using DADA2 in the QIIME2 software (Version QIIME2-202202) to generate amplicon sequence variants (ASVs) [21]. The DADA2 analysis was run with truncation lengths of 0, a maxEE value of 2.0, and the consensus method for chimeras. Taxonomic annotation of each ASV was performed using a pre-trained Naive Bayes classifier with the classify-sklearn algorithm in QIIME2, against the Silva 138.1 database [22,23]. The absolute abundance of ASVs was normalized using a standard of sequence number corresponding to the sample with the least sequences. The subsequent result analysis is based on the normalized data.

One-way ANOVA with Duncan’s multiple comparisons test, performed using SPSS 29.0, was employed to analyze changes in enzyme activities, intestinal villi data, and dominant microbiota across different developmental stages. The Results are presented as mean ± SE; *p* < 0.05 denoted statistical significance. Figures were generated in GraphPad Prism 8.0.2.

## 3. Results

### 3.1. External Morphological Changes in Sichuan Bream

**Figure 2:**
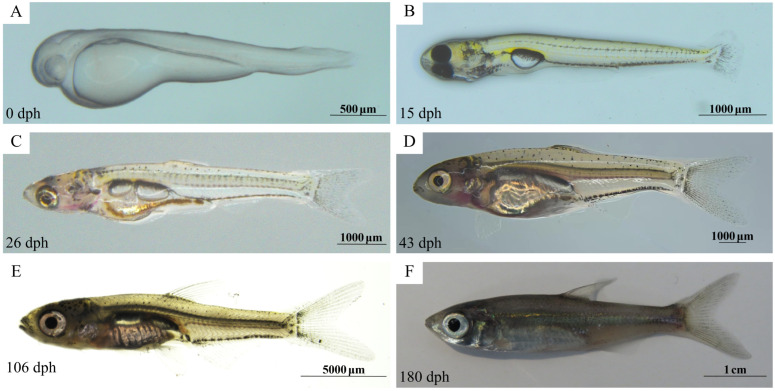
Different developmental stages of Sichuan bream. (**A**) G1 stage. (**B**) G2 stage. (**C**) G3 stage. (**D**) G4 stage. (**E**) G5 stage. (**F**) G6 stage.

At stage G1, larvae appeared colorless and transparent without pigment deposition. Mouths remained unopened, with discernible eye vesicles but no distinct organs (Figure 2A). By G2, larvae initiated feeding and developed xanthophore pigmentation while retaining transparency, revealing intestinal tracts. A single-chambered swim bladder exhibited prominent melanization on its surface and ocular structures. Stellate melanophores were distributed laterally, and the caudal fin displayed a fan-shaped morphology (Figure 2B). G3 larvae displayed semi-transparent heads and translucent abdomens with visible intestines. The cardiac outline was observable beneath the opercular margin. Eyes protruded with yellowing internal capsules. A two-chambered swim bladder developed—elliptical anterior chamber and rod-shaped posterior chamber. The caudal fin notched centrally (forked), while pectoral, anal, and dorsal fins initiated formation (Figure 2C). At G4, scales emerged dorsally, while the head and abdomen became opaque. All fins were fully developed, with intensified melanization forming a distinct lateral line (Figure 2D). G5 subadults achieved complete squamation with reduced body transparency and a darkened lateral line (Figure 2E). G6 sexually mature adults exhibited near-complete body opacity (Figure 2F).

### 3.2. Intestinal Morphological Development

**Figure 3:**
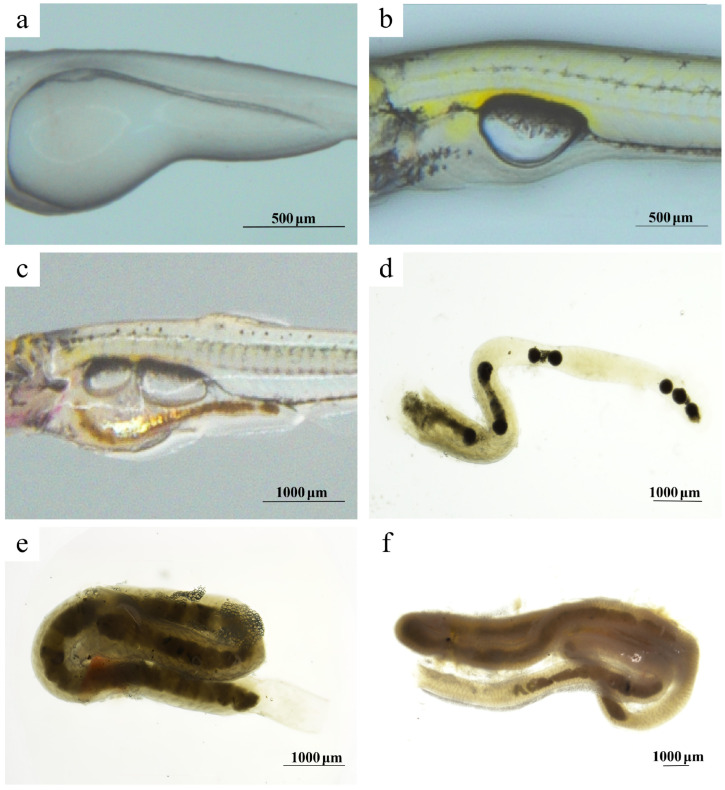
Intestinal morphological change in Sichuan bream. (**a**) Intestinal tract at G1 stage. (**b**) Intestinal tract at G2 stage. (**c**) Intestinal tract at G3 stage. (**d**) Intestinal tract at G4 stage. (**e**) Intestinal tract at G5 stage. (**f**) Intestinal tract at G6 stage.

At G1, no discernible digestive tract had formed (Figure 3a). By G2, the larval intestine became patent, exhibiting an S-shaped bend in the anterior-mid region while the posterior section remained straight (Figure 3b). During G3, the S-shaped curvature of the intestinal tract intensified (Figure 3c). Dissection of G4 juveniles revealed an intestine with a broad anterior and tapered posterior, forming two bends in an N-shaped configuration (Figure 3d). In G5 subadults, intestinal bends became more developed and compactly arranged (Figure 3e). By G6, adults displayed multiple intestinal flexures (Figure 3f).

**Figure 4:**
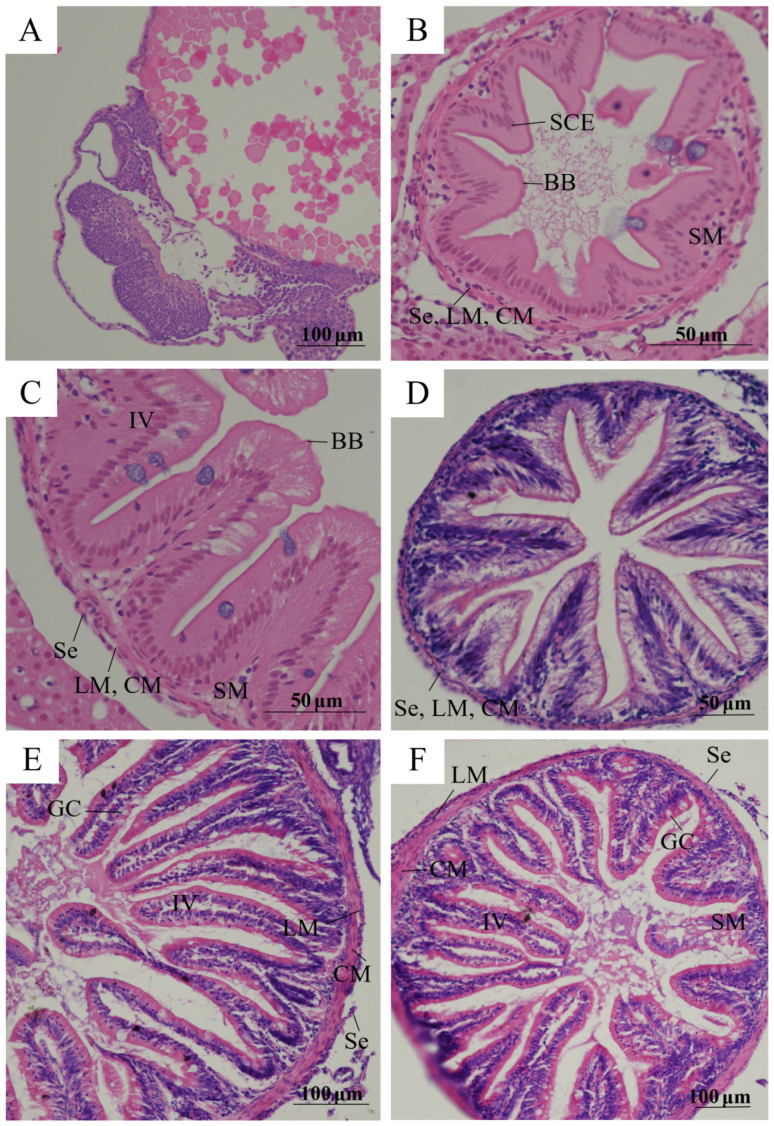
Intestinal histology structure of Sichuan bream. (**A**) Transversal intestinal histology structure of whole larvae at G1 stage. (**B**) Transversal intestinal tissue structure of whole larvae at G2 stage. (**C**) Transversal intestinal histology structure of whole larvae at G3 stage. (**D**) Transverse intestinal histology structure of mid-foregut region of juvenile fish at G4 stage. (**E**) Transverse intestinal histology structure of mid-foregut region of subadult fish at G5 stage. (**F**) transverse intestinal histology structure of mid-foregut region of adult fish at G6 stage. BB: brush border; IV: intestinal villi; SCE: simple columnar epithelial cells; SM: submucosa; CM: circular muscle; LM: longitudinal muscle; Se: serosa; GC: goblet cell.

As shown in Figure 4, the digestive tract of Sichuan bream at stage G1 remained undeveloped (Figure 4A). Histological examination of G2 larvae revealed four distinct intestinal layers from lumen outward: mucosa, submucosa, muscular layer, and serosa. The mucosa contained villi with visible brush border at their apical ends and consisted of tightly packed simple columnar epithelial cells (Figure 4B). During subsequent development, goblet cells became evident among the columnar epithelial cells (Figure 4C–F). The submucosa, primarily composed of connective tissue, contained abundant blood vessels and cells. The muscular layer comprised an inner circular muscle (thicker) and outer longitudinal muscle. The outermost serosa appeared extremely thin, formed by connective tissue enveloped by peripheral mesothelium.

Furthermore, intestinal villus height progressively increased across five developmental stages (Table 1), with successive increases of 200.09%, 2.32%, 97.34%, and 25.03%. Significant differences (*p* < 0.05) in villus height were observed between most stages. Villus width also exhibited an overall increasing trend, showing significant elevation (*p* < 0.05) at G3 compared to G2 before stabilizing thereafter.

### 3.3. Dynamics of Digestive and Immune Enzyme Activities

**Figure 5:**
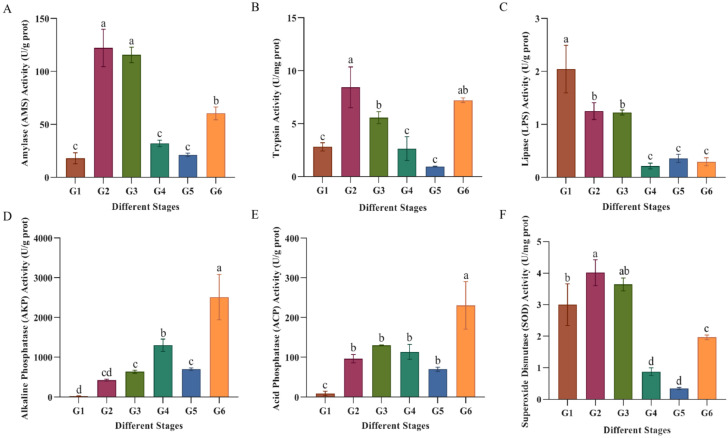
Changes in enzyme activity of Sichuan bream at different developmental stages. (**A**) Changes in AMS activity. (**B**) Changes in trypsin activity. (**C**) Changes in LPS activity. (**D**) Changes in AKP activity of Sichuan bream. (**E**) Changes in ACP activity. (**F**) Changes in SOD activity. Different lowercase letters indicate significant differences (*p* < 0.05).

Among digestive enzymes, AMS and trypsin activities peaked at stage G2, subsequently declined, then increased significantly at G6 (*p* < 0.05) (Figure 5A,B). LPS activity was highest at G1 and gradually decreased, stabilizing at low levels thereafter (Figure 5C). The activities of immune enzymes (AKP, ACP and SOD) in Sichuan bream generally showed a trend of increase-decrease-increase (Figure 5D–F). All three immune enzymes exhibited significant upregulation at G6 (*p* < 0.05).

### 3.4. Overview of Sequencing Results

Analysis of 16S rRNA data from all 36 samples yielded 2,798,413 high-quality sequences (mean ± SD: 77,734 ± 22,223; range: 29,238–113,605). DADA2 denoising generated 8320 amplicon sequence variants (ASVs) (Table 2). Newly hatched larvae (C1) exhibited the highest number of gut-specific ASVs. Water-unique ASVs showed an initial decrease followed by an upward trend, peaking in W6. Only 11 ASVs were shared across all Sichuan bream gut microbiota groups (C_1_–C_6_), while 59 ASVs were common to all water microbiota groups (W_1_–W_6_). Merely 6 ASVs were shared between all gut microbiota and water microbiota groups (Figure S1).

### 3.5. Diversity Analysis

**Figure 6:**
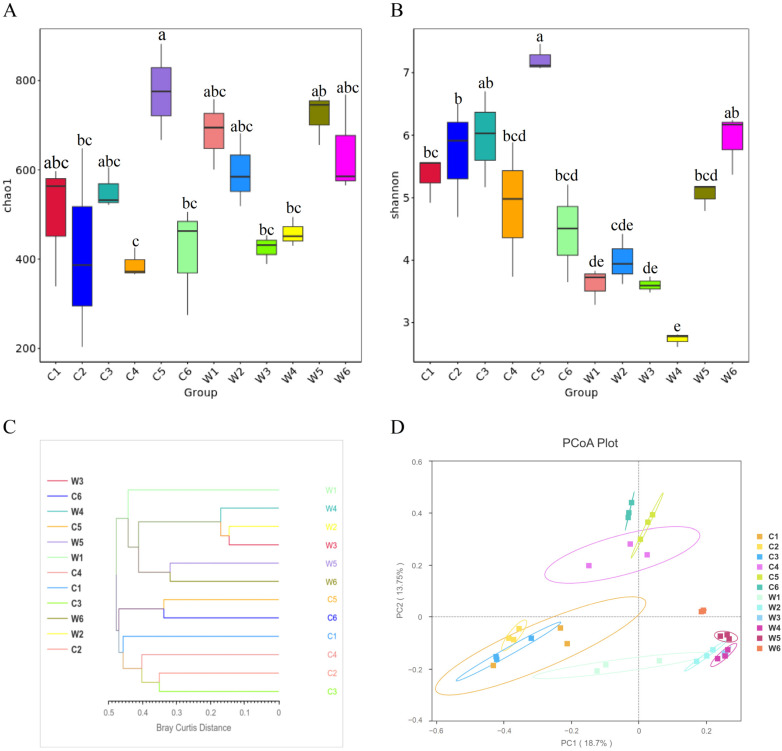
Comparison of α and β diversity among different groups. (**A**) Chao1 Index among all groups. (**B**) Shannon Index among all groups. (**C**) UPGMA (Bray–Curtis) cluster tree analysis of all groups. (**D**) PCoA (Unweighted Unifrac) of all groups. Different lowercase letters indicate significant differences (*p* < 0.05).

Alpha diversity analysis revealed significantly increased Shannon and Chao1 indices in the gut microbiota of C5 (*p* < 0.05), marking the highest values across all developmental stages (Figure 6A,B). This indicates greater species richness and more even distribution in the gut microbiota of Sichuan bream subadults. For rearing water microbiota, both indices initially decreased then increased, with the Shannon index in W4 being significantly lower than other stages (*p* < 0.05). Notably, no significant differences existed between gut microbiota and contemporaneous water microbiota Chao1 indices (*p* > 0.05). However, gut microbiota Shannon indices were significantly higher than concurrent water microbiota (*p* < 0.05), except between C6 and W6, suggesting that the species distribution of the gut microbiota is more uniform.

In the beta diversity analysis, the samples were hierarchically clustered using the Unweighted Pair-group Method with Arithmetic Mean (UPGMA) based on the Bray–Curtis distance. The similarity clustering situation between intestinal and water samples was observed (Figure 6C). The results showed that all water samples clustered into one branch, while all intestinal samples clustered into another. Principal coordinate analysis (PCoA) based on the Unweighted Unifrac distance was performed on the gut and rearing water bacterial communities. The results also demonstrated separate clustering of the gut and rearing water microbiota. Within the gut bacterial community, the C4 group served as a watershed. The distances between the C1, C2, and C3 groups were relatively close, as were the distances between the C4, C5, and C6 groups (Figure 6D). However, PERMANOVA (Permutational Multivariate Analysis of Variance) revealed no significant difference between the gut microbiota and the contemporaneous rearing water microbiota at any individual developmental stage (*p* > 0.05) (Table 3).

### 3.6. Microbiota Succession

**Figure 7:**
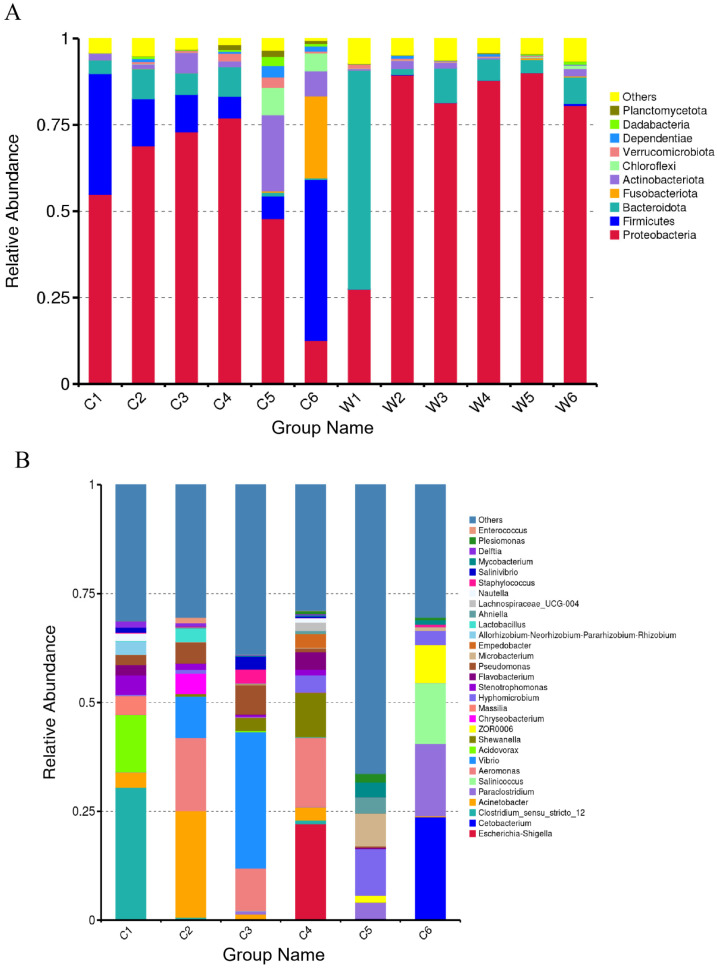
Relative abundance (%) of dominant microbiota. (**A**) Dominant phyla in the gut and rearing water microbiota. (**B**) Dominant genera in the gut microbiota.

Phylum-level analysis revealed Proteobacteria, Firmicutes, Bacteroidota, Actinobacteriota, and Fusobacteria were identified as the dominant phyla in both the gut and rearing water microbiota of Sichuan bream (Figure 7A). Within gut microbiota, Proteobacteria prevailed from larval to subadult stages (C_1_–C_5_) while Firmicutes dominated adults (C6), with Bacteroidota peaking at C2, Actinobacteriota attaining peak relative abundance at C5, and Fusobacteria reaching maximal abundance at C6 (Figure S2). Rearing water microbiota showed Bacteroidota dominance in W1, shifting to Proteobacteria (>80% relative abundance) in W2–W6. The top 10 gut microbial genera included *Escherichia/Shigella*, *Acinetobacter*, *Aeromonas*, *Vibrio*, *Acidovorax*, *Shewanella*, *Paraclostridium*, *Salinicoccus*, *Clostridium_sensu_stricto*, and *Cetobacterium* (Figure 7B). *Acidovorax* and *Clostridium_sensu_stricto* abundances were highest in C1 before significantly decreasing (*p* < 0.05), whereas *Paraclostridium*, *Salinicoccus*, and *Cetobacterium* remained low through C1–C5 before peaking at C6 (*p* < 0.05). *Escherichia*/*Shigella*, *Acinetobacter*, *Aeromonas*, *Vibrio*, and *Shewanella* exhibited unimodal abundance patterns: *Acinetobacter* and *Aeromonas* peaked at C2 (*p* < 0.05), *Vibrio* at C3 (*p* < 0.05), and *Escherichia*/*Shigella* and *Shewanella* at C4 (*p* < 0.05) (Table S1).

### 3.7. The Contribution of Rearing Water Microbiota to the Gut Microbiota

**Figure 8:**
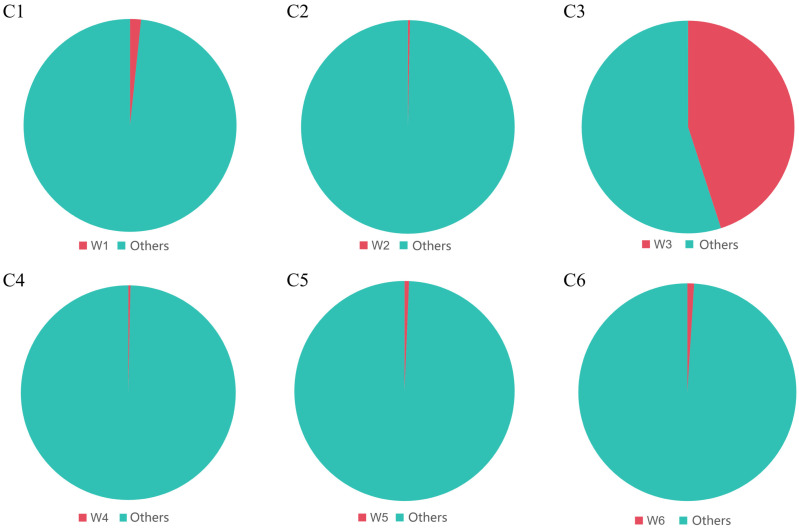
Contribution of rearing water to gut microbiota of Sichuan bream.

To explore the contribution of the rearing water to the gut microbiota of Sichuan bream, a microbial source tracking analysis was conducted across different developmental stages. The intestinal tract was designated as the sink and the rearing water as the source. The portion of the gut microbiota not originating from the rearing water is labeled “others”. The results revealed that the microbiota in the C1, C2, C4, C5, and C6 groups exhibited minimal contributions from their contemporaneous water sources (1.67%, 0.33%, 0.33%, 0.67%, and 1.00% from W1, W2, W4, W5, and W6, respectively). In stark contrast, a significantly higher proportion (45.00%) of the microbiota in the C3 group was derived from the W3 water source (*p* < 0.05) (Figure 8).

## 4. Discussion

After hatching, the intestinal tract and other organs of Sichuan bream gradually develop and mature [19]. According to the intestinal segmentation methods for cyprinid fish described by Wang et al. [24] and Yan et al. [25], the intestine of Sichuan bream was divided into the foregut, midgut, and hindgut. The segment from the end of the esophagus to the first bend was defined as the foregut, the segment from the first bend to the second bend as the midgut, and the segment after the second bend up to the anus as the hindgut. Our histological observations of the foregut revealed that the height of the intestinal villi increased significantly (*p* < 0.05) during most developmental stages of Sichuan bream, thereby increasing the intestinal surface area and enhancing nutrient absorption efficiency [26]. Furthermore, the densely distributed goblet cells within the mucosal layer secrete mucus, which protects the mucosal layer [27]. The dense network of blood vessels and abundant blood cells in the submucosa efficiently transports nutrients absorbed by the intestine throughout the body [28]. The muscular layer not only propels chyme but also exerts pressure on it, ensuring thorough contact with the intestinal mucosa and thereby increasing nutrient absorption efficiency.

The activity of digestive enzymes largely determines the nutritional digestibility of ingested feed by animals. The main digestive enzymes in fish are amylase, lipase, and trypsin. LPS activity in Sichuan bream peaked during the newly hatched larval stage. A possible reason is that yolk lipids provide substantial energy for larvae during the endogenous nutritional stage [29]. Accordingly, the content of phospholipase A2, an enzyme used for digesting yolk lipids, is high at this time [30,31]. This also indicates that the ontogeny of digestive enzymes in newly hatched Sichuan bream larvae is regulated by their own genes [32,33,34,35]. The overall trends in amylase and trypsin activities were similar, both increasing significantly and peaking during the G2 stage. This increase may be related to the stimulation of food intake, suggesting that after initial feeding, Sichuan bream larvae require high digestive capacity to adapt to exogenous feeding [36]. Trypsin activity decreased significantly during the G3 stage, indicating that the dietary transition in Sichuan bream affected its protein digestion capacity, possibly due to a lower protein content in the formulated feed compared to *Artemia*. Subsequently, the activity of AMS and trypsin increased significantly during the adult stage (G6). Combined with the overall high levels of the three digestive enzymes during the larval stage, this suggests that both the larval and adult stages of Sichuan bream require substantial nutrients for growth and development, warranting enhanced feed provision and monitoring during these periods. Furthermore, trypsin activity was significantly higher than LPS and AMS activity throughout the developmental process (*p* < 0.05), indicating that Sichuan bream possesses a high capacity for protein digestion.

Studies have shown that Proteobacteria, Firmicutes, and Fusobacteriota are the dominant phyla in the gut microbiota of freshwater fish [37,38]. In this study, analysis of the dominant phyla and the top 10 abundant genera in the intestine of Sichuan bream revealed that Proteobacteria, Firmicutes, Fusobacteriota, Actinobacteriota, and Bacteroidota are the main components of its gut microbiota. Among these, Proteobacteria dominated from groups C1 to C5, but decreased significantly in the adult stage (C6), while the relative abundances of other phyla increased. Li et al. [39] also found in their research a mutually exclusive pattern between Proteobacteria and phyla such as Firmicutes and Fusobacteriota, with distinct functional characteristics. Compared to phyla like Firmicutes, Fusobacteriota, and Bacteroidota, Proteobacteria contain fewer genes for carbohydrate degradation and short-chain fatty acid production, while encoding more virulence factors and antibiotic resistance genes. Proteobacteria encompass many pathogenic bacteria [40], such as the genus *Vibrio*, which was dominant in group C3 and exhibits significant pathogenicity in fish [41,42,43]. Additionally, the genus *Acinetobacter*—which dominated in the C2 group—includes several members that confer multi-drug resistance and are considered pathogenic in fish [12,44].

Throughout the development of Sichuan bream, beneficial bacteria are also present. They maintain the balance of the gut microbial community and host health through interactions such as nutrient competition and inhibitory metabolite production [45]. The relative abundance of Bacteroidota increased significantly (*p* < 0.05) and peaked in group C2. Studies suggest that microbes within Bacteroidota can assist the host in polysaccharide absorption [46]; the high abundance of Bacteroidota at this stage, when Sichuan bream begins feeding exogenously, may be related to its nutritional absorption needs. In group C5, the relative abundance of Actinobacteriota increased significantly (*p* < 0.05), while that of Proteobacteria decreased significantly (*p* < 0.05). Research indicates that members of Actinobacteriota can inhibit pathogens by secreting antimicrobial substances, directly participating in fish immune defense mechanisms [47,48]. In Sichuan bream, the high abundance of Actinobacteriota in group C5 might have exerted inhibitory effects on some pathogenic bacteria within Proteobacteria. Firmicutes evolved from a subdominant community in group C1 to the dominant community in group C6 (Figure 3 and Figure 4). This pattern of change is similar to the developmental pattern of the gut microbiota in largemouth bass [20]. The genus *Clostridium_sensu_stricto* within Firmicutes and the genus *Acidovorax* within Proteobacteria had the highest relative abundances in group C1. However, their abundances decreased significantly (*p* < 0.05) as development progressed, suggesting these genera might originate from the maternal source or the water environment during fertilization but cannot establish long-term colonization [45]. The relative abundances of *Cetobacterium* (within Fusobacteriota) and the genera *Paraclostridium* (within Firmicutes) and *Salinicoccus* (within Firmicutes) all increased significantly (*p* < 0.05) in group C6. The dominance of these bacterial communities in group C6 may primarily relate to enhanced energy metabolism during this period. Existing studies show that Firmicutes can enhance host nutrient absorption efficiency [49], and the genus *Cetobacterium* is significantly positively correlated with the fish’s carbohydrate metabolism capacity [50]. Moreover, researchers have discovered that the genus *Cetobacterium* is also related to the synthesis of vitamins B-12 [51].

Proteobacteria and Bacteroidota dominated the water environmental microbiota of Sichuan bream. Among water samples, Bacteroidota had the highest relative abundance in group W1. In groups W2-W6, Proteobacteria became the absolutely dominant phylum (relative abundance > 80%). This dynamic change is likely related to the influence of feeding practices on water quality after Sichuan bream began exogenous feeding. SourceTracker analysis revealed that the water microbiota contributed to the composition of the Sichuan bream gut microbiota. Specifically, 45.00% of the gut microbiota in group C3 originated from the water, significantly higher than in other groups. Similarly, in a study tracing the source of gut and water microbiota in *Acrossocheilus fasciatus*, it was found that 28.67% of the gut microbiota in the juvenile stage came from water, while the contribution of rearing water to the gut microbiota was significantly lower in other stages [52]. Group G3 Sichuan bream larvae transitioned from *Artemia* to formulated feed. We speculate that microbiota from the water could enter the intestine along with ingested feed. Studies have shown that environmental microorganisms always enter the host gut via dietary pathways. Depending on feeding habits, specific fish species can only introduce specific environmental microorganisms into their intestines [53,54,55]. For Sichuan bream under artificial rearing conditions, group G3 larvae transitioned from *Artemia* to formulated feed. Feed particles in the water adsorb surrounding microbiota. When Sichuan bream consumes these particles, microbiota attached to them have more opportunities to enter the digestive tract. Furthermore, research shows that the gut microbiota is not randomly acquired from the environment but is selectively retained by the host [56]. Researchers studying wild fish populations in the Yangtze River found that as the host develops, ingested transient microorganisms are screened by the host’s intestinal environment, with only a small portion forming stable symbiotic relationships with the host [57].

## 5. Conclusions

The results indicate that the intestinal structure of Sichuan bream progressed from a straight tube through S-shaped and N-shaped configurations to multiple flexures. Histology revealed its four foregut layers (mucosa, submucosa, muscular layer, serosa), coupled with increasing foregut villus height. These adaptations enhance nutrient digestion and absorption, supporting developmental demands. Key digestive enzyme profiles necessitate intensified feed management during larval and adult stages. The sustained high trypsin activity (*p* < 0.05) indicates a high capacity for protein digestion; diet formulations with appropriately elevated protein levels may be beneficial, pending validation in growth trials. Gut microbiota analysis showed Proteobacteria dominating C1–C5, succeeded by Firmicutes in C6, with Bacteroidota (C2), Actinobacteriota (C5), and Fusobacteriota (C6) peaking sequentially. Rearing water microbiota contributed to the gut microbiota, peaking at G3 when Sichuan bream completed dietary transition to formulated feed. Subsequent host selection established symbiosis with only a minority of this microbiota. Thus, gut microbiota dynamics are intrinsically linked to developmental stages in Sichuan bream. This study did not investigate the initial water microbiota prior to fish contact or the impact of feed-derived bacteria on the gut microbiota. These aspects can be explored in future research.

## Figures and Tables

**Table 1:** Changes in intestinal villus height and width of Sichuan bream in different developmental stages.

Item	G2	G3	G4	G5	G6
Intestinal villus height (μm)	35.55 ± 4.03 ^d^	110.09 ± 10.34 ^c^	112.64 ± 2.32 ^c^	222.28 ± 17.88 ^b^	277.91 ± 29.96 ^a^
Intestinal villus width (μm)	31.62 ± 2.97 ^b^	57.84 ± 5.39 ^a^	52.77 ± 6.22 ^a^	62.15 ± 9.59 ^a^	61.55 ± 1.38 ^a^

Note: Different lowercase letters indicate significant differences (*p* < 0.05).

**Table 2:** Results of 16S rRNA data analysis.

Samples	Sequences	ASV	Phylum	Genus
C1	88,071	1201	29	275
C2	97,019	1075	29	242
C3	92,869	1172	31	310
C4	102,759	819	23	219
C5	86,507	1196	26	194
C6	98,766	702	23	139
W1	81,800	1034	28	191
W2	64,539	988	28	199
W3	53,742	741	26	142
W4	65,417	790	26	140
W5	66,381	1104	29	199
W6	34,932	1212	30	250

**Table 3:** PERMANOVA test.

Group	Df	SumsOfSqs	MeanSqs	F.Model	R2	Pr (>F)
C1-W1	1 (4)	1.00976 (0.68566)	1.00976 (0.17142)	5.89067	0.59558 (0.40442)	0.1
C2-W2	1 (4)	1.09801 (0.60022)	1.09801 (0.15006)	7.31736	0.64656 (0.35344)	0.1
C3-W3	1 (4)	0.97607 (0.2321)	0.97607 (0.05803)	16.82129	0.80789 (0.19211)	0.1
C4-W4	1 (4)	1.07663 (0.70314)	1.07663 (0.17579)	6.12465	0.60492 (0.39508)	0.1
C5-W5	1 (4)	1.31138 (0.12585)	1.31138 (0.03146)	41.67944	0.91243 (0.08757)	0.1
C6-W6	1 (4)	1.02808 (0.66869)	1.02808 (0.16717)	6.14985	0.60591 (0.39409)	0.1

## Data Availability

The raw data presented in the study are openly available in NCBI Gene Expression Omnibus database (BioProjectID: PRJNA1310037).

## Supplementary Materials

The following supporting information can be downloaded at: https://www.mdpi.com/article/10.3390/ani15233431/s1, Figure S1: Venn diagram. (A) Venn diagram of all groups. (B) Venn diagram of gut microbiota of Sichuan bream. (C) Venn diagram of water microbiota. Figure S2: Relative abundance of dominant intestinal microbiota in Sichuan bream at different developmental stages (phylum level). Different lowercase letters indicate significant differences (*p* < 0.05). Table S1: The dynamic changes of the top ten intestinal microbial genera of Sichuan bream at different developmental stages. Note: 0 indicates a value < 0.005; ± is standard deviation, values < 0.005 are also omitted. Different lowercase letters indicate significant differences (*p* < 0.05).
