# Supplementary figures and images for: Intestinal Development Patterns and Gut Microbiota Colonization Dynamics in Sichuan Bream (Sinibrama taeniatus)
Source: Animals (Basel). 2025 Nov 28;15(23):3431. doi: 10.3390/ani15233431 (PMC12691183; doi:10.3390/ani15233431)

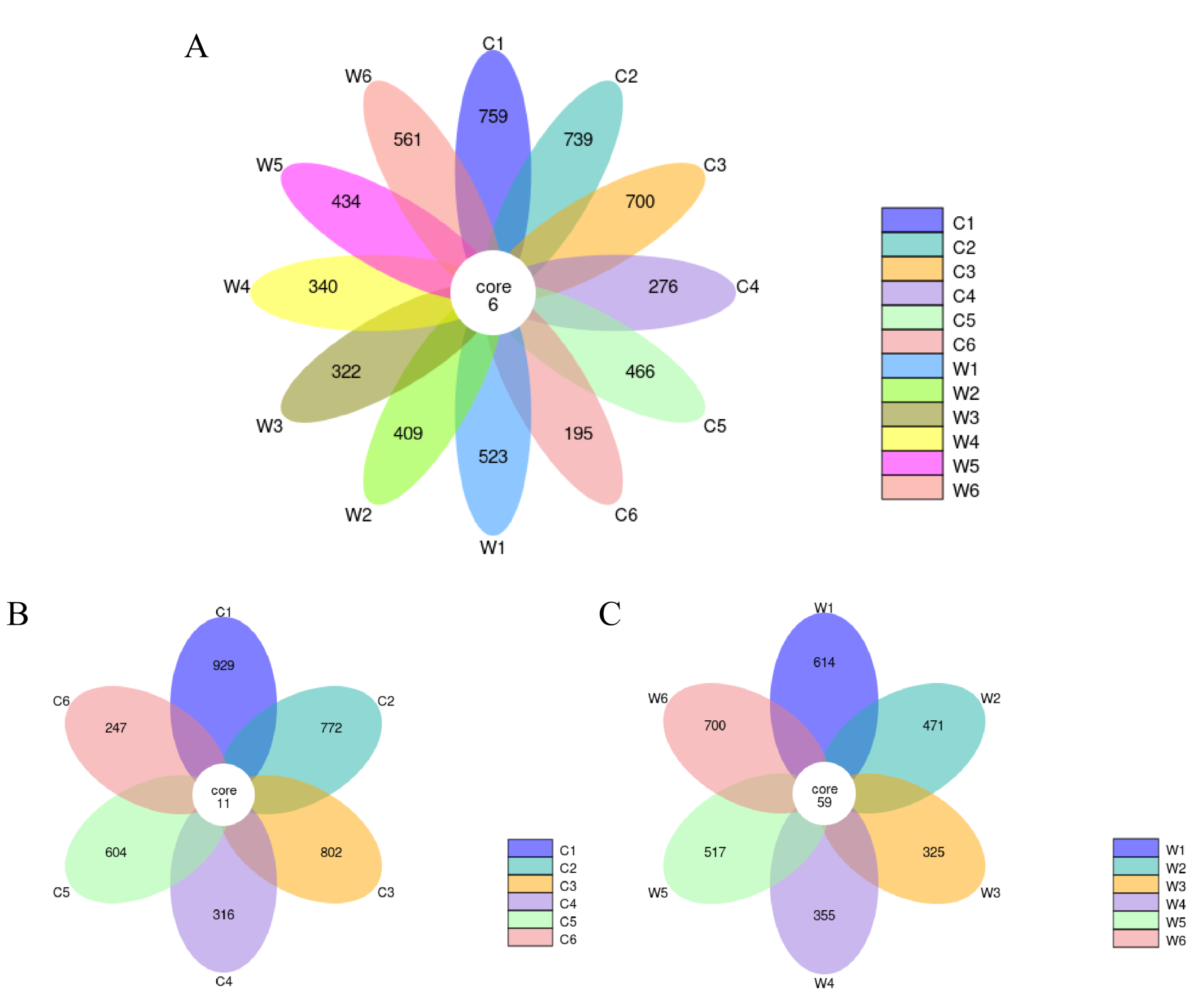

Supplement: Supplementary file 1 [file animals-15-03431-s001.zip › Figure S1.tif]

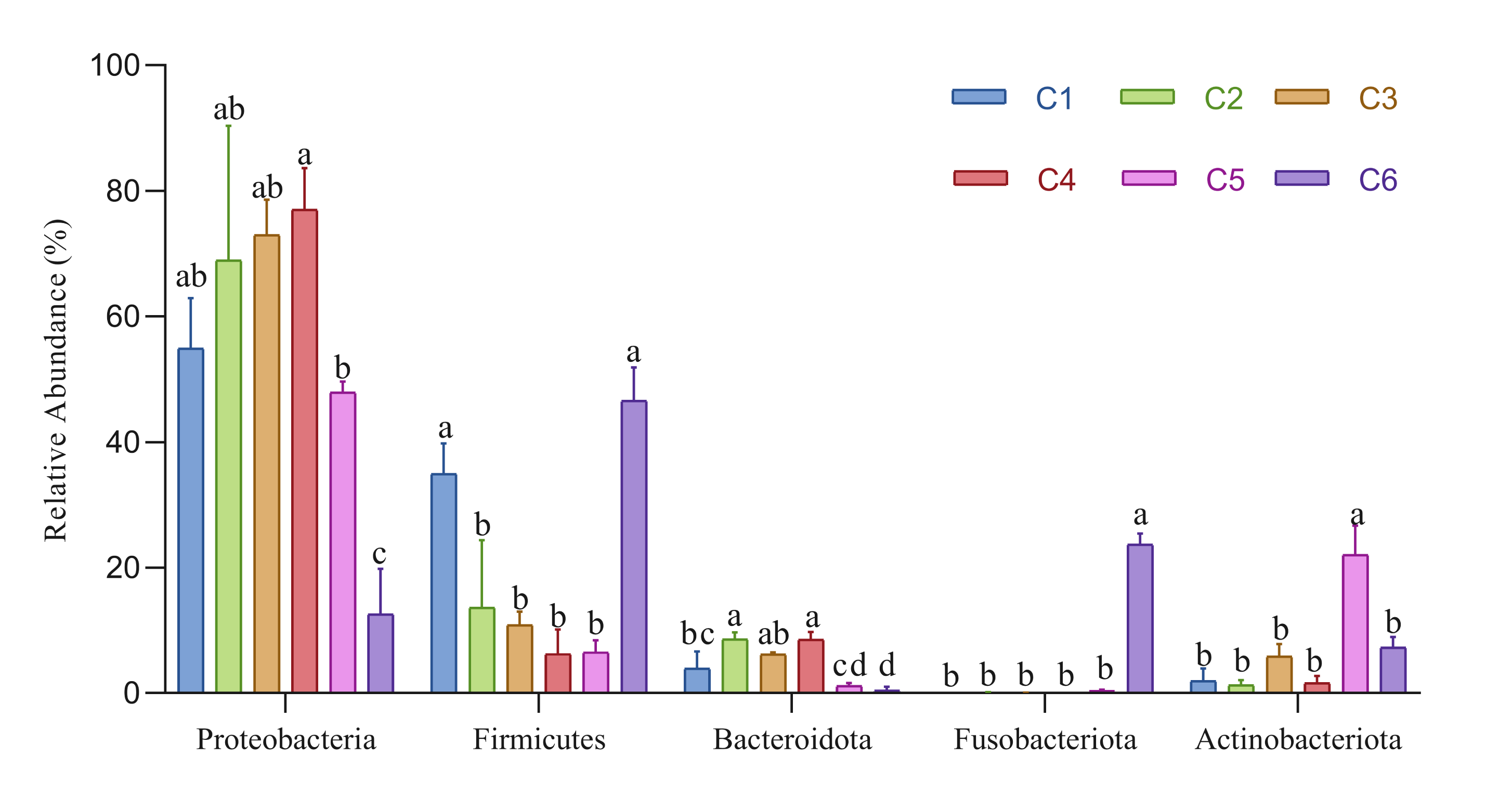

Supplement: Supplementary file 1 [file animals-15-03431-s001.zip › Figure S2.tif]
